# Comparing the Impact of Topical Application of Human Milk and Chlorhexidine on Cord Separation Time in Newborns

**DOI:** 10.12669/pjms.321.8223

**Published:** 2016

**Authors:** Fatemeh Abbaszadeh, Zanab Hajizadeh, Mohammad Jahangiri

**Affiliations:** 1 Fatemeh Abbaszadeh, Department of Midwifery, Kashan University of Medical Science, Kashan, Iran; 2 Zanab Hajizadeh, BS of Midwifery, Beheshti Hospital, Kashan University of Medical Science, Kashan, Iran; 3 Mohammad Jahangiri, Neonatologist, Department of Pediatrics and Neonatology, Kashan University of Medical Science, Kashan, Iran

**Keywords:** Human Milk, chlorhexidin, Separation Time, Umbilical Cord

## Abstract

**Objectives:**

The best umbilical cord care after birth is a controversial issue. Aim of this research was to compare the effect of topical application of human milk and chlorhexidin on cord separation time in newborns.

**Methods:**

One hundred seventy four neonates attending from hospitals affiliated to Kashan University of Medical Sciences were included. Newborns from birth were randomized to two groups. In group mother’s milk, mother will rub her own milk to cord stump two times a day. chlorhexidin (group 2) were applied to the umbilical stump every 12 hours. The time to umbilical cord separation and any discomfort such as infection, hemorrhage, and discharge and odor were documented. Data was analyzed by SPSS software. Independent Samples t-Test, χ^2^, Fisher were used in this study.

**Results:**

Results showed a significant statistical difference between cord separation time in two groups and the mean cord separation time in the human milk group (7.15±2.15days) was shorter than the chlorhexidin group (13.28±6.79 days). In addition, a significant correlation was found between Signs of infection (discharge, redness, swelling and odor) in both groups.

**Conclusions:**

Topical application of breast milk on umbilical cord care leads to quick cord separation time and can be used as easy, cheep, non injury methods for umbilical cord care.

## INTRODUCTION

After delivery, the umbilical cord serves no purpose. The necrotic tissue of the umbilical cord is a potential site for infection^1^ immediately after birth by bacterial contaminants.^2^ The umbilical cord is an important bacterial colonization site, which may occasionally lead to neonatal infection such as omphalitis and sepsis. Corrected umbilical cord care is important to prevent infections in neonatal period.^2^-^4^ While there is a general agreement about the ‘clean’ technique for cutting the cord using a sterile cutting instrument (blade or scissors) and clean hands to avoid infection, there is less agreement on what is the best care of the cord stump.^5^,^6^

WHO recommends that chlorhexidine is the preferred agent if an antiseptic is to be used on the cord, for example in settings in which it might be strategic to use antiseptic applications to the cord to discourage the use of dung or other unclean substances.^7^ Chlorhexidine is a broad spectrum antiseptic which acts effectively on gram positive and negative bacteria and viruses including HIV.^8^ Novak et al. showed that using antiseptics on the cord stump can lead to reduction of leukocytes which are necessary for cord separation through decreasing bacterial colonization^8^-^10^ and the resulted delay in cord separation can increase unnecessary postpartum care and visits.^11^ Delay in cord separation can increase the risk of infections.^12^,^13^

Furthermore, one of the methods which is used for caring umbilical cord is topical application of breast milk that has been used in Kwazula-Natal, some societies of Kenya and some areas of Turkey; and today by getting aware of complications of other methods and useful properties of breast milk, it has been used again.^14^ Human milk is a source of two classes of major growth factors, namely the transforming growth factors alpha and beta (TGF-A and TGF-B) and the insulin-like growth factors 1 and 2 (IGF-1 and IGF-2).^15^ These growth factors promote muscle and cartilage repair and wound healing.^16^ TGF-A and TGF-B are involved in normal cell activities such as embryonic development, cell proliferation, and tissue repair. IGF-1 has pronounced anabolic and wound-healing characteristics. It slows catabolism, and it is the only growth factor that can stimulate muscle growth and repair by itself.^14^ Conflicting with this condition, cord treatment may destroy the resident flora and facilitate invasion by pathogenic, and it may delayed umbilical cord separation time^6^, other hand in developing countries where mothers receive postnatal visits by midwives, problems with the cord often determines the number of visits. If cord separation and healing are delayed, as when some antimicrobials are used the cost of postnatal care may increase unnecessarily.^6^,^14^

Milk is always available and it can be used as an easy, cheap and non invasive way for the cord care, it is important to explore the possibility of using human milk topically to protect infants from umbilical cord infections in developing countries. A search of the literature found no study that compared human milk with chlorhexidine solution. We decided to compare the impact of two regimens of cord care, topical use of human milk and chlorhexidine solution on the cord separation time in newborns of hospitals of Kashan.

## METHODS

In a clinical trial, 174 neonates (87 neonates in each group) well-babies were enrolled from hospitals affiliated to Kashan University of Medical Sciences in Kashan city, Central Iran that is located in Isfahan Province. Inclusion criteria for the study were: Newborns with gestational age 42-37 weeks; Apgar score higher than 7 at minute 1and 5; rupture of membranes for no longer than 12 hour. criteria for exclusion were: Infants with perinatal asphyxia, respiratory distress, metabolic disease, and any other problems that requiring immediate refer to neonatal Intensive Care Unit immediate; Infants with immediate need of evaluation and treatment; Newborns with any congenital disorder or disease; Infants of mothers with postpartum fever, mastitis, Urogenital infection; If the mother does not live in the city of Kashan [live in Rural). The umbilical cord of all the newborns was cut in sterile condition in the delivery room and no antiseptic agent was used on the stump. All the neonates were born in baby-friendly hospitals with rooming-in in one hundred percent of the cases and all the mothers had complete vaccination against tetanus.

The infants were randomly assigned into two groups of cord care regimens: Group 1 received topical application of human milk to the umbilical stump from three hours after birth every 12 hours (2 times a day) to 2 days after the umbilical cord separation. Group 2 receive chlorhexidine solution, by a sterile gauze or swab, to the umbilical stump from three hours after birth and continued every 12 hours until two days after umbilical cord separation. All infants born from 12:00 AM Saturday to 11:59 PM Friday of the first week were randomly selected to the chlorhexidine group, then the next week’s group to breast milk. The weekly selection was based on avoiding connection among mothers, because there is no possibility of separating the mothers to place restrictions in two groups.

All mothers received information on the importance of umbilical cord care and signs of its infection during a session for three hours after birth. In this research we used chlorhexidine solution 4% (Hydrex, Ecolab Co, Germany). In order to make sure of the presence of the microorganisms, cultures of the cord stump were collected using sterile swabs within the first three hours after birth. Samples were taken to the laboratory for analysis by the microbiologist. Mothers in two group received forms to record the exact time of mothers’ milk or chlorhexidine application during the day. Moreover, the mothers in both groups were asked to daily record infection signs of the cord stump in special forms. signs of local infection Including: discharge, redness, inflammation, and swelling of the umbilical cord. All mothers were asked to continue completing the forms until two days after the cord separation. Follow-up on mothers by telephone calls and during visit at home was done. In order to make sure the presence of the infection, researchers visited umbilical cord within the first three hours after birth at hospital and days 3, 7 and 2 days after umbilical cord separation at home and in cases of delay in cord separation or any cord related adverse events such as blood leakage, mucoid discharge or granuloma formation, the newborn was immediately visited by neonatologist.

SPSS software (version11.5) was employed to analyze the data. Differences in means were analyzed using independent sample t-test. Chi-square test and Fisher’s exact test was also applied to nominal variables. P-value of less than 0.05 was regarded to be significant.

The study protocol was approved by the local research council in Kashan University of Medical Sciences. All women were free to participate and they were assured of confidentiality of their personal information, also this study was approved by the research ethic committee in Kashan University of Medical Sciences.

## RESULTS

**Fig.1:**
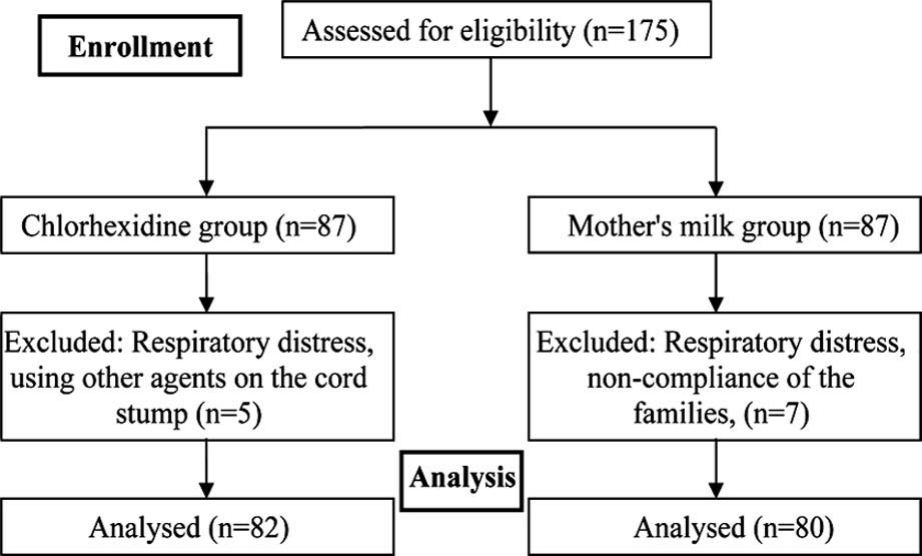
Flow Chart.

Initially, 174 neonates were studied during 2010-2012. After follow up, 5 neonates in the chlorhexidin group and 7 neonates in the mother’s milk group were excluded due to several reasons such as respiratory distress, using other agents on the cord stump and non-compliance of the families, and finally the data from 162 newborns was analyzed. Fig.1 shows the loss of samples.

Demographic and perinatal characteristics of the neonates in the two groups of mother’s milk and topical chlorhexidine solution are shown in Table-I. These characteristics were not significantly different between the two groups except for the duration of membrane rupture. Mean time of membrane rupture was 2.22±2.7 hours in the topical chlorhexidine group and 2.61±1.52 hours in the mother’s milk method which showed a statistically significant difference. Since the main consequence of prolonged rupture of membrane is infection, and the first culture of the cord stump taken within the first three hours after birth showed no significant difference between the groups, it seems unlikely that this factor has a significant effect on the study results.

**Table-I:** Absolute and relative frequency distribution of demographic and perinatal characteristics in Chlorhexidine and mother’s milk groups.

Demographic and Perinatal characteristics	Chlorhexidine group N=82 No. (Percentage)	Mother’s milk group N=80 No. (Percentage)	P value
*Maternal age, y*			
15-24	27(32.9)	19(23.8)	P=0.7
25-29	29(35.4)	31(38.8)	
30-34	16(19.5)	17(21.3)	
35>-	10(12.2)	13(16.3)	
Mean ± SD	27.20±5.14	5.30±28.29	
*Education of mother*			
Illiterate & Primary school	12(14.6)	16(20)	P=0.820
Middle school	16(19.5)	16(20)	
High school	39(47.6)	33(41.3)	
College education	15(18.3)	15(18.8)	
*Occupational status*			
Unemployed	75(91.5)	69(86.3)	P=0.211
Employed	7(8.5)	11(13.8)	
*Sex of newborn*			
Male	46(56.1)	46(57.5)	P=0.491
Female	36(43.9)	34(42.5)	
*Weight of newborn*			
2000-2999	10(12.2)	14(17.5)	P=0.575
3000-3999	65(79.3	58(72.5)	
4000-5000	(8.5)	8(10.0)	
Mean ± SD	3384.14±450.87	3306.38	
*Time of rupture of membranes*			
Birth	25(30.4)	45(56.3)	
0.5-6 hours before birth	49(59.8)	30(37.5)	
6-12 hours before birth	8(9.8)	5(6.3)	
Mean ± SD	2.75±2.31	2.61±1.52	

Table-II shows the mean time of cord separation in topical chlorhexidine group and mother’s milk group. The shortest cord separation time was three days in group topical human milk, and the longest was 53 days in group chlorhexidine.

**Table-II:** Comparison of statistical indices related to the cord separation time in Chlorhexidine and Mother’s milk groups.

Variable	Chlorhexidine group	Mother’s milk group
Mean &Standard deviation(day)	13.28±6.79	7.14±2.15
Minimum time of cord separation(day)	4	3
Maximum time of cord separation(day)	53	14
P value	P<0.001	

Signs of local infection showed no significant difference between the two groups, (Table-III). The results showed no significant different between the two groups regarding frequency of physician visits (P=0.167) or drug side effect and P=0.267, respectively).

**Table-III:** Absolute and relative frequency of local infection signs in chlorhexidine and mother’s milk groups.

Infection signs	Chlorhexidine group	Mother’s milk group	P value

	No. (Percentage)	No. (Percentage)	
Discharge	34(41.5)	33(44)	
Redness	17(20.7)	19(25.3)	
Inflammation	9(11)	3(4)	

## DISCUSSION

The present research is the first study to compare the effect of topical application of human milk and chlorhexidin on umbilical cord stump separation time. Human milk may accelerate the complicated process of the cord separation through polymorphonuclear leukocytes present at the cord, proteolysis enzymes and other available immunologic compounds.^14^ Breast milk has a lot of immunologic and anti-infective agents and colostrums contains significantly quantities of complement components that act as natural antimicrobial agents and is also equipped with protective factors that provide specific and non specific passive immunity.^17^

In most of samples, cord separation times of five to ten days were found in 67.5%, 34.5% in group topical human milk and chlorhexidine, respectively. In topical human milk group, the umbilical cords were detached within the normal period (between 5-15 days following the birth) documented in literatures.^18^ This result is matched with Gulsen Vural and Sezer Kisa (2006), who examined the incidence of omphalitis among three groups, each using a different type of newborn cord care: povidone-iodine, dry care, and topical human milk. They reported that 62.7% of the newborns in the study had cord separation times of 6 to 10 days.^14^ Azza A, (2011) in his study reported that, More than half (58%) of neonates in the breast milk group their umbilical cord stump separated on 5th and 6th day after birth.^18^ Mehrus et al has reported that cord separation was faster in the human milk neonates than in the alcohol group. In this regard, almost half of human milk group babies had their cord fallen off at the 3rd-4th day of age and 23 (46%) of them had their cord fallen off at the 5th-6th day of age.^19^ This difference between various studies regarding the separation time of umbilical cord stump might be due to intervening factors such as local circumstances and community as (sanitation, weather, and humidity).

Statistical analysis of our findings showed that mean cord separation time in the newborns receiving topical chlorhexidine solution is significantly longer than mother’s milk groups group (13.28±6.7 vs. 7.14±2.15 days respectively). In addition, the longest time of cord separation was 53 days in chlorhexidine group and 14 days in mother’s milk group. So far there have been no studies on the effect of topical application of human milk and chlorhexidin on umbilical cord stump separation time. Mullany et al (2005) investigated the effect of topical chlorhexidine treatment on cord separation in neonates of south Nepal and found that mean time to cord separation is significantly longer in chlorhexidine use (5.35 days) in comparison to dry cord care (4.24 days) and the possibility of cord separation after day 7 was 3.6 times higher in the chlorhexidine group.^8^ Study of Ahmadpoor, et al. showed the mean cord separation time in the human milk group was significantly shorter than alcohol, silver sulfadiazine, and dry cord care groups (P=0.001).^20^ Study of Gulsen Vural and Sezer Kisa showed babies in the topical human milk group had shorter cord separation times(7.0±2.0day) than those in the povidone-iodine, cord separation occurred at a mean of 9.9±3.3 days.^14^ In addition, Azza A, in his study reported that, Umbilical cord separation time occurred early for neonates in the breast milk group Vs neonates in the distilled water group (5.60 + 1.04 & 7.92 + 1.08 days, respectively). (P< 0.001).^18^ Moreover, Zupan et al concluded that caring the umbilical cord with antiseptics can lead to delayed cord separation and natural dry care is as effective and safe as application of antibiotics and antiseptics.^21^ It seems that using antiseptics is associated with longer cord separation time.^22^

In addition, a significant correlation was not found between signs of infection (discharge, redness and swelling) in both groups. No cases of granuloma formation and sepsis were found in the present study and none of the neonates needed to be hospitalized. In other words, mother milk can be effective as chlorhexidine (broad-spectrum antibiotics) in reducing signs of infection of cord. This result is matched with study by Ahmadpour-Kacho et al.^20^

## CONCLUSION

Use of topical application of human milk on umbilical cord care was associated with shorter cord stump separation time than in topical chlorhexidin. Breast milk also reduced incidence of of infection, as much as topical chlorhexidin. Generally human milk is always available and it can be used as an easy, cheap and non invasive way for the cord care.
